# Effects of amino acid composition in pig diet on odorous compounds and microbial characteristics of swine excreta

**DOI:** 10.1186/s40781-017-0153-5

**Published:** 2017-12-11

**Authors:** Neeraja Recharla, Kihyun Kim, Juncheol Park, Jinyoung Jeong, Yongdae Jeong, Hyunjeong Lee, Okhwa Hwang, Jaehyoung Ryu, Youlchang Baek, Youngkyun Oh, Sungkwon Park

**Affiliations:** 1 Department of Food Science & Biotechnology, Sejong University, 209 Neundong-ro, Gwangjin-gu, Seoul, 05006 South Korea; 2 National Institute of Animal Science, RDA, Wanju, 55365 South Korea

**Keywords:** Ideal protein, Amino acid, Growth performance, Microbial communities, Short chain fatty acid (SCFA), Pyrosequencing, Volatile odorous compounds (VOS)

## Abstract

**Background:**

Major amino acids in pig diets are Lys, Met, Thr, and Trp, but little is known about the requirements for the other essential amino acids, especially on odorous compounds and microbial characteristics in feces of growing-finishing pigs. To this end, different levels of amino acid composition added to diets to investigate the effects of amino acid composition on microbial characteristics and odorous compounds concentration.

**Methods:**

A total eight (*n* = 8) barrows (Landrace × Yorkshire × Duroc) with an average bodyweight of 89.38 ± 3.3 kg were individually fed diets formulated by Korean Feeding Standards 2007 (old version) or 2012 (updated with ideal protein concept) in metabolism crates with two replication. After 15-day adaptation period, fresh faecal samples were collected directly from pigs every week for 4 weeks and analysed for total volatile fatty acids (VFA), phenols and indoles by using gas chromatography. The nitrogen was determined by Kjeldahl method. Bacterial communities were detected by using a 454 FLX titanium pyrosequencing system.

**Results:**

Level of VFA tended to be greater in 2012 than 2007 group. Among VFAs, 2012 group had greater (*p* < 0.05) level of short chain fatty acids (SCFA) than control.Concentration of odorous compounds in feces was also affected by amino acid composition in pig diet. Levels of ammonium and indoles tended to be higher in 2012 group when compared with 2007 group.Concentration of phenols, p-cresol, biochemical oxygen demand, and total Kjeldahl nitrogen, however, were lower (*P* < 0.05) in 2012 treatment group compare to 2007. The proportion of *Firmicute* phylum were decreased, while the *Bacteriodetes* phylum proportion increased and bacterial genera including*Coprococcus, Bacillus, and Bacteroides* increased (*p* < 0.05) in 2012 compare to 2007 group.

**Conclusion:**

Results from our current study indicates that well balanced amino acid composition reduces odor by modulating the gut microbial community. Administration of pig diet formulated with the ideal protein concept may help improve gut fermentation as well as reduce the odor causing compounds in pig manure.

## Background

Consumption of meat and meat products have been increased dramatically probably due to their higher incomes in Korea [1]. Each Korean ate 9.5 kg of beef & veal, 28.4 kg of pork meat, 14.4 kg of poultry meat and 0.2 kg of sheep meat [2]. Especially, Korean meat consumers prefer for pork belly and Boston butt which contain high fat [3].

Production cost can be reduced by improving the market weight. To this end, The specific standards for nutritional requirements for swine were established early in 1940s by National Research Council (NRC) and in Korea, the National institute of Animal Science revised a feeding standard of swine in 2012, which adapted the ideal protein concept for pig production [4].

The source of odour from pig farming facilities is due to generation of large amounts of urine, feces and fermentation of this mixture during storage [5–7]. The odour of pig excreta is the result from anaerobic microbial degradation of the components of the feed in gut, especially some odours from proteins are more toxic and offensive sensation than the carbohydrates [8, 9]. Many types of odorants have been identified from samples of fresh and rotten swine manure [10] and these were classified into four main groups, namely straight- and branched-chain volatile fatty acids, indoles and phenols, ammonia and volatile amines, and the volatile sulfur compounds [6, 11]. Acetic acid, propionic acid and butyric acids are most common volatile fatty acids (VFA) produced from microbial conversion of dietary residues. The straight chain- VFA derived from both plant fibre and protein microbial degradation and the branched chain-VFA produced by anaerobic fermentation of amino acids such as leucine, isoleucine, lysine [7, 12]. Sulfur-containing volatile compounds such as hydrogen sulphide, dimethyl sulphide, carbonyl sulphide are originates from microbial degradation of amino acid substrates like cysteine and cysteine [13]. Aromatic compounds such as phenols, *p*-cresol, indoles and skatole are by-products of tyrosine and tryptophan degradation. These compounds are formed in the gut microflora and also during anaerobic storage of swine manure [7]. Nitrogenous compounds, ammonia and volatile amines are produced from protein degradation. Ratio of amino acids is an important element that affects the generation of odorous compounds in swine excreta [9, 14]. Several studies have been reported that decreasing crude protein (CP) level can be reduced faecal odorous compounds [15–17].

The term ideal protein can be defined as the protein which containing the minimum quantity of essential amino acids with maximum utilization to meet the exact nutritional amino acid requirements [18]. It refers to determine the required amounts of amino acids relative to lysine for maintenance, protein accretion, and growth performance of pigs [19, 20]. Munks et al. [21] first initiated the ideal protein concept to poultry for production of chicken egg. Later, Mitchell [22] was determined the requirement of amino acids for different animal species. In 1981, British agriculture research council (ARC) was first formulated feed based on ideal protein concept [19]. Since then, various new effective formulations have been developed based on ideal protein concept, such as Institute National de la recherche agronomique (INRA) in 1984 [23], NRC in 1988 [24], and Wang & Fuller in 1989 [18].

Until now, there is no reports on the effects of feed formulated based on the ideal protein concept on odorous compound generation from pig farm. Therefore, the objective of this study was to compare the excretion compounds and bacterial community from faeces of pigs fed diets formulated either by 2007or 2012 Korean feeding standards of swine.

## Methods

### Animals

A total eight (*n* = 8) barrows ({(Landrace × Yorkshire) × Duroc}] with an average bodyweight of 89.38 ± 3.3 kg were used for this study. These pigs were randomly allocated into two diet (2007 & 2012) groups. Pigs were housed individually in metabolism cages and the temperature was maintained at 25 ° C. The experiment was performed two replications. All procedures involving animals were approved by the National Institute of Animal Science Animal Care and Use Committee.

### Diets

Feeds were formulated based on 2007 & 2012 Korean feeding standard for swine. The form of the feed was ground form, ingredient composition for diets were given in Table 1 and nutrient content are shown in Table 2. The Feed were fed in a limited manner, approximately 1.5 kg was fed to each pig per twice a day at 10.00 am and 16.00 pm. Pigs had free access to water throughout the experiment.

**Table 1:** Ingredient composition of experimental diets

Ingredient (%)	2007 (Control)	2012 (Treatment)
Cornstarch	40	40
Corn	40	40
Beet pulp	11.069	10.224
Soybean meal	1.077	.
Wheat bran	0.318	0.275
Soybean oil	4.425	5.166
Salt	0.17	0.174
^a^DCP	1.404	1.447
Lysine	0.563	0.761
Methionine	0.064	0.132
Arginine	.	0.139
Histidine	0.038	0.084
Isoleucine	0.169	0.314
Leucine	0.091	0.315
Phenylalanine	0.142	0.19
Threonine	0.223	0.347
Tryptophan	0.069	0.106
Valine	0.179	0.325
Total	100	100

^a^ *DCP* digestible crude protein

**Table 2:** Nutrient content in experimental diets

	2012	2007
^a^DE, kcal/kg	3400	3400
^b^ME, kcal/kg	3360	3359
^c^CP, %	8.43	7.822
Ca, %	0.45	0.45
P, %	0.4	0.4
Na, %	0.1	0.1
lysine, %	0.76	0.64
methionine, %	0.23	0.17
arginine, %	0.32	0.22
histidine, %	0.24	0.21
isoleucine, %	0.46	0.34
leucine, %	0.76	0.58
phenylalanine, %	0.38	0.36
threonine, %	0.5	0.4
tryptophan, %	0.14	0.11
valine, %	0.53	0.41

^a^ *DE* Digestible energy, ^b^ *ME* Metabolizable energy, ^c^ *CP* Crude protein

### Sampling collection

After 15-day adaptation period, fresh faecal samples were collected directly from pigs every week for 4 weeks. The 40 ml of each sample was taken into 50 ml tube and then stored in a refrigerator for further analysis.

Blood sample was collected from the vein with using syringe. After collecting the blood, 2 ml of collected blood was transferred into a tube containing anticoagulant (K2 EDTA 5.4 MG tube BD Vacutainer REF 367856) for haematology analysis, and a tube containing anticoagulant (Lithium Heparin tube BD Vacutainer REF 367884) 2 ml of blood was added and 8 ml of blood serum was stored in a serum separation tube (BD SST II AVANSAT REF 367953).

### Growth performance study

Initial and final body weight of pigs in each group were measured to calculate the average daily gain (ADG). Feed intake and excretion (faeces & urine) were recorded to determine the feed efficiency by calculating feed conversion ratio (FCR).

### VFA (volatile fatty acids) analysis

VFA analysis were performed by gas chromatography (GC) (6890 N, Agilent, Santa Clara, CA, USA), equipped with an HP - INNOWax column and a flame ionization detector.

To analyze the VFA, 5 ml of slurry sample, 25% phosphoric acid solution and 1 ml of saturated mercury solution (Sigma-Aldrich, St. Louis, Mo., USA) were taken into a 15 ml tube and then the solution was centrifuged at 3134 x g for 20 min. Thereafter, 1 ml of the supernatant was centrifuged at 13,800 x g for 10 min and filtered through a 0.2 μm filter (Whatman, Uppsala, Sweden). The filtrates were placed in 2.0 ml GC vials (Agilent, Santa Clara, Calif., USA) to measure the concentration of volatile fatty acids by GC.

0.2 μLwas the sample injection volume with a split ratio of 10:1.The temperature of the oven started from 80 °C, and then initially increased by 20 °C per minute and kept at 120 °C for 2 min, then the temperature was upgraded to 205 °C by increasing 10 °C per minute, finally, it was maintained at 205 °C for 2 min. The injection and detection ports were maintained at 250 °C.

### VOC (volatile odorous compounds) analysis

The concentrations of phenols and indoles in slurry sample were quantitatively measured by Gas Chromatography. For this measurement, the slurry sample was centrifuged for 20 min at 3134 x g at 20 °C. 4 ml of the supernatant liquid was added to the 20 ml glass bottle which containing 4 ml of chloroform and 60 μm 4 M of sodium hydroxide solution. Then the mixture was centrifuged at 20 °C at 3134 x g for 20 min and then 2.0 ml of the chloroform layer was transferred to a GC vial to analyze the phenols and indoles by using GC equipped with flame ionization detector (FID) and DB-1 column (30 m × 0.25 mm × 0.25 μm, Agilent, Santa Clara, Clara, CA, USA). 0.2 μLwas the sample injection volume with a split ratio of 5:1. The temperature of oven was programmed as follows: initially started from 40 °C and held for 5 min and it upgraded to 230 °C by increasing 10 °C per minute. Then 230 °C was kept for 2 min. The injection and detection ports were maintained at 250 °C. The nitrogen was determined by Kjeldahl method [25].

### Microbial community analysis

The bacterial communities of pig slurry samples were analysed by PCR amplification based on 16S rRNA gene sequences by using the 27F and 518R primer pair.

### PCR amplification for bar-coded pyrosequencing

In the first step, genomic DNA from slurry sample was isolated by Fast-DNA Spin Kit (MP Bio, Santa Ana, CA, USA) according to the manufacturer’s instruction manual. Humic acid, which is inhibiting PCR amplification was removed using the Power-Clean DNA Clean-Up Kit (MP Bio, Santa Ana, CA, USA). The amplification conditions of the PCR were followed: initially 1 cycle at 95 °C for 5 min and then 30 cycles of 30 s at 95 °C, 30 s at 55 °C, 72 °C for 30 s, and finally one cycle was performed at 72 °C for 7 min.

### Pyrosequencing and data analysis

Pyrosequencing analysis was performed by Chunlab (Seoul, Korea) using the 454 FLX titanium System (Roche, Pleasanton, CA, USA). Sequencing reads of samples were assigned each sequence read to specific sample by their endemic bar codes. PCR primer sequences, barcode, and linker were then removed from the original sequence analysis. For the next analysis, the pyrosequencing reads were selected based on quality filtering process, which are containing more than 300 base pair with an average quality score of more than 25. EzTaxon-e database were used with the BLAST search tool to perform taxonomic alignment of bacterial high-quality sequence reads. Sequences that could not be matched the EzTaxon-e database, which is at 97% species level were used to a second-order UCHIME program to identify unrealistic sequences. Operational taxonomic units (OTUs) were generated using a CD-HIT program at with a similarity level of 97%. The Shannon-Weaver diversity index, Chao1 richness index and Goods library coverage were calculated using the Mothur program.

### Statistical analysis

Concentration of odorous compounds data were analysed by using statistical analysis system [26] with using analysis of variance of GLM (General linear model). Significance difference among means of control and treatment groups were compared using Duncan’s multiple range tests [27]. Statistical analysis was also performed using Pearson’s correlation coefficient to determine the relationship between concentrations of odorous compounds and the relative abundance of bacterial species. Probability value less than 0.05 (*P* < 0.05) was considered for all measured variables.

## Results and discussion

### Growth performance

Although effects of CP levels on the concentration of odorous compounds and gut microbiota have been well established [17], little is known about the essential amino acids based diet impact on the microbial community changes in swine gut. Therefore, this study was performed to evaluate the impact of administration of diet formulated based on the ideal protein concept on growth performance, odorous compounds production, and gut microbial communities in pigs.

There was no significant difference in feed intake, BW, ADG, FCR, or amount of total faeces and urine excretion between treatment groups (Table 3). Lopez et al. [28] demonstrated that the ideal protein based diet improved the feed efficiency and carcass leanness in gilts but does not affect the growth performance. Similarly, Hong et al. [29] were also reported that feeding low energy and low protein diets with adequate amino acid supplements had no negative effects on growth performance of growing-finishing pigs. Other studies on piglets and grower-finisher pigs showed that dietary CP levels can be reduced by formulating pig diets with supplementary amino acids including Lys, Met, Thr, Trp, Phe, His, Val, Ile, and Leu without compromising their growth performance and feed efficiency when feeds were formulated by ideal protein concept [30, 31]. Hence, utilization of these supplements will reduce feed costs and improve profits for swine farmers as well as provide other benefits on environment by reducing N wastage and concentration of odours.

**Table 3:** Growth performance of pigs^a^

	2012	2007	^f^SEM	*p*-value
Total feces, ^b^DM, g	263	264	20.02	0.97
24 h total urine, g	2708	3085	137.13	0.18
Feed intake, g	3000	3000	0	–
Initial ^c^BW, 3.30, kg	89.38	89.38	3.05	1
Final BW, 4.13, kg	105	106	2.42	0.85
^d^ADG, g/day	1.12	1.19	0.05	0.51
^e^FCR	2.74	2.57	0.12	0.50

^a^Means of 8 pigs per treatment with two replicates

^b^ *DM* Dry Matter, ^c^ *BW* Body Weight, ^d^ *ADG* Average Daily Gain, ^e^ *FCR* Feed Conversion Ratio^f^ *SEM* Standard errors of the means

### Effects of dietary treatment on volatile odorous compounds (VOC)

The effects of pig diets formulated either by 2007 or 2012 feeding standard on levels of VOC are shown in Table 4. Phenolic volatile compounds are one of the major causes for odour in fresh slurry [32, 33]. Phenol was not detected in faecal samples of 2012 treatment group. Biochemical oxygen demand (BOD) levels were significantly decreased (*P* < 0.05) in 2012 group when compared with 2007 group. Levels of p-cresol and phenols were lower (P < 0.05) and pH tended to be lower (*P* = 0.07) in faeces from pigs fed 2012 diet compare to those fed 2007 diet. Other compounds including phenole, indole, skatole, total Kjeldahl nitrogen (TKN), and N also tended to decrease in 2012 group, suggesting odorous compound levels were decreased across the board by supplementation of pig diets formulated based on the ideal protein concept.

**Table 4:** Effects of dietary treatment on VOCs^a^

VOC, ppm	2012	2007	*p*-value
phenol	0	0.14	0.34
p-Cresol	15.29	23.33	0.02
Indole	1.72	1.43	0.19
Skatole	0.08	0.22	0.44
^b^phenols	15.29	23.47	0.03
^c^Indoles	1.81	1.65	0.43
pH	7.08	6.79	0.07
^d^BOD	6985	9255	0.06
^e^TKN	920	983	0.06
NH4-N	239	180	0.07
N	0.301	0.327	0.08
C	0.758	0.811	0.66
S	0.536	0.503	0.37
H	11.645	11.602	0.88

^a^VOC, volatile odorous compound; means of 8 pigs per treatment with two replicates

^b^Phenols = Phenol + p-Cresol, ^c^Indoles = Indole + Skatole, ^d^ *BOD*, Biochemical Oxygen Demand, ^e^ *TKN* Total Kjeldahl Nitrogen

Canh et al. [34] have demonstrated that low levels of CP with essential amino acid supplements reduces nitrogen excretion and ammonia emission without compromising growth performance of growing-finishing pigs. Reduced CP content with adequate amino acids supplementation also showed reduced urinary energy loss as well as greater growth performance [35]. In the current study, CP levels included either in 2007 or 2012 diet were not statistically different, but adequate and appropriate ratio of amino acids were provided for 2012 diet (Table 2). There was abundant evidence that pigs fed ideal protein based diets showed maximum utilization of absorbed protein for maintenance and for tissue protein accretion (for growth) with minimum nitrogen (total N) excretion [36–38]. Reduced N excretion in pig slurry is closely associated with decrease in the odour emission from pig facilities [39]. Total Kjeldahl nitrogen (TKN) is the sum of organic nitrogen, ammonia (NH3), ammonium (NH4) and therefore to calculate total nitrogen, concentration of nitrate-N and nitrite-N needs to be determined and added to TKN [40]. Total N and TKN levels, interestingly, tended to be lower but ammonium nitrogen (NH_4_-N) was higher in 2012 group than in 2007 group in our study. Since conversion of TKN into certain type of N depends on the type of protein present in the sample, especially what fraction of the protein is composed of nitrogenous amino acids, further study will be necessary to evaluate the intermediate processes of N conversion.

### Effects of dietary treatment on volatile fatty acids (VFA) concentration

Administration of diets formulated by 2007 or 2012 feeding standard affected the levels of VFA (Table 5). Compare to pigs fed 2007 diet, those fed 2012 diet numerically increased levels of all kinds VFAs analysed in the current study, including acetic acid, propionic acid, butyric acid, valeric acids, iso-butyric acid and iso-valeric acid, and branched chain fatty acids (BCFA). Level of SCFA, especially, was significantly increased (*P* < 0.05) in 2012 group (759 ppm) when compared with 2007 group (708 ppm). Dietary proteins and carbohydrates are substrates for microbial fermentation in large intestine [41]. Fermentation of undigested proteins may produce potentially harmful end products including phenols, indoles, ammonia and BCFA [17]. In our current study, however, levels of these odorous compounds tended to be lower by supplementation of 2012 diet, which was formulated with well-balanced amino acid composition. Other studies showed that adequate amino acid supplements or addition of fermentable carbohydrates in pig diets helped reduce malodorous chemical generation and also improved gut health by conversion of fermentable carbohydrates into SCFA by microbes residing in the large intestine [42, 43].

**Table 5:** Effects of dietary treatment on VFA concentration

^a^VFA, ppm	2012	2007	^d^SEM	*p*-value
Acetic acid	483	443	14.34	0.17
Propionic acid	170	163	5.52	0.71
Butyric acid	83	81	3.12	0.08
Valeric acid	23	21	0.71	0.56
Iso-butyric acid	22	21	1.08	0.47
Iso-valeric acid	39	36	2.18	0.24
^b^SCFA	759	708	22.46	0.02
^c^BCFA	62	57	3.27	0.50

^a^ *VFA*, volatile fatty acids; means of 8 pigs per treatment with two replicates

^b^ *SCFA*, Short Chain Fatty Acid = Acetic acid + Propionic acid + Butyric acid

^c^ *BCFA*, Branched Chain Fatty Acid = Iso-butyric acid + Iso-valeric acid

^d^ *SEM* Standard errors of the means

### Effects of dietary treatment on microbial communities

Bacterial communities in animal gut play important roles in degradation of undigested protein and carbohydrates, influence the gut healthiness by inhibiting colonization of pathogens, and maintain gut microbial ecosystem, which is essential to maintain normal gut physiology [44]. Results of 16S rDNA sequence analysis performed by using 454 FLX titanium multiplex bar-coded pyrosequencing system to determine the microbial community changes in the faecal samples of pigs fed 2007 or 2012 based diet are shown in Table 6. The total number of valid readings obtained 13,264 in the 2012 specification and 14,018 in the 2007 specification. Among these, 635 and 672 OTUs were detected in 2012 and 2007 group, respectively. Richness and diversity indices of communities are also showed in Table 6. Significant difference was not appeared in any item, but OUT counts tended to be lower in 2012 group when compared with 2007 group. Bacterial communities of 2007 manure samples showed more Jackknife numbers than those of 2012 group. Values of Shannon–Weaner index (H) were 3.95 and 4.11 in 2012 and 2007, respectively. There was no significant difference in Simpson’s index (D) or Goods library coverage between two diet groups.

**Table 6:** The pyrosequencing data of bacterial communities in pig feces^a^

	2012	2007
Total valid reads	13,264	14,018
^b^OTUs	635	672
Ace	815	824
Chao 1 shared richness	774	777
JackKnife	829	849
Shannon–Weaner index (H)	3.95	4.11
Simpson’s index (D)	0.1	0.1
Goods library coverage	98	99

^a^Four samples from each treatment were analyzed for pyrosequencing data

^b^OUT Operational Taxonomic Units, Shannon-Weaver (diversity index), Chao1 (richness index) and Goods library coverage were calculated using the Mothur package

**Fig. 1:**
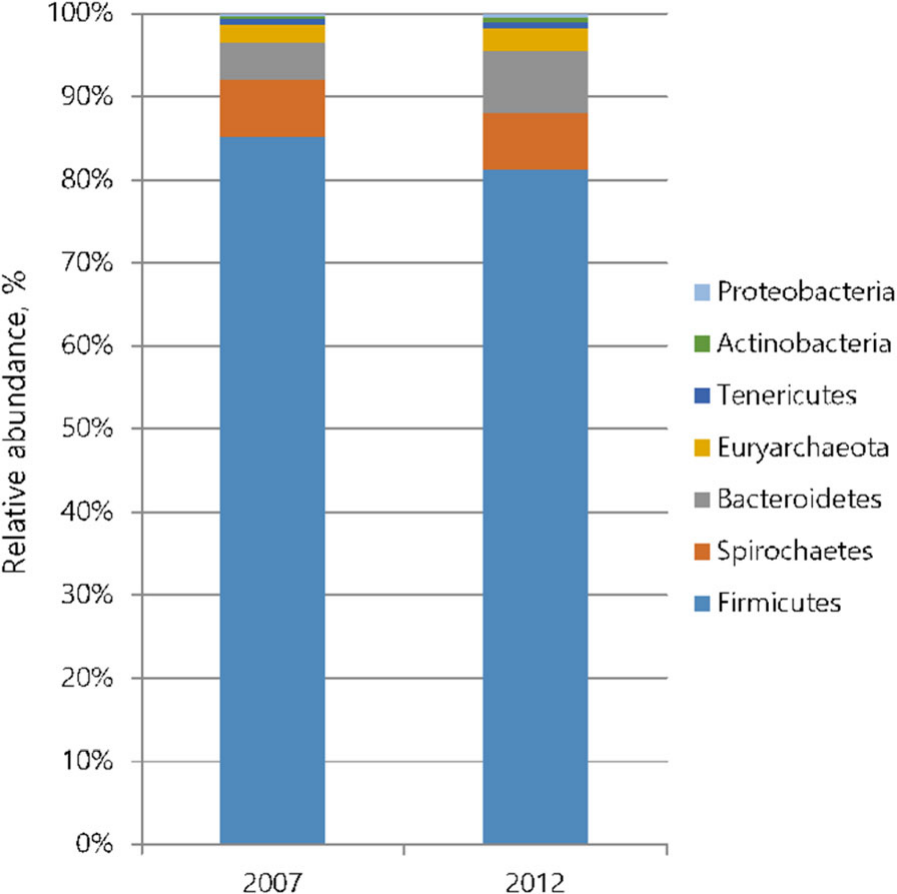
Bacterial taxonomic compositions of phylum level^*^. ^*^Four samples from each treatment were analyzed for composition of phylum level

Bacterial taxonomic compositions in phylum level of feces from pigs fed 2007 or 2012 are shown in Fig. 1. *Firmicutes* and *Bacteroidetes* are major microbial phyla in mamals gut [45, 46]. Concomitant to these reports, results from our current study showed that *Firmicutes* are most dominant phyla in both groups, followed by *Spirochaetes,* and *Bacteroidetes*. At phylum level, the proportion of *Firmicutes* decreased, while that of *Bacteroidetes* were significantly increased (*P* < 0.05) in 2012 group. The microbiota in the gut produce SCFA such as acetate, propionate, and butyrate by fermenting nutrients from pig diet. Pigs fed 2012 diet showed a greater level of SCFA, which is known to positively influence the odor concentration [8]. The *Bacteroidetes* mainly produces propionate and acetate, therefore this phylum has been linked with the higher levels of total SCFAs in 2012 group [47, 48].

**Fig. 2:**
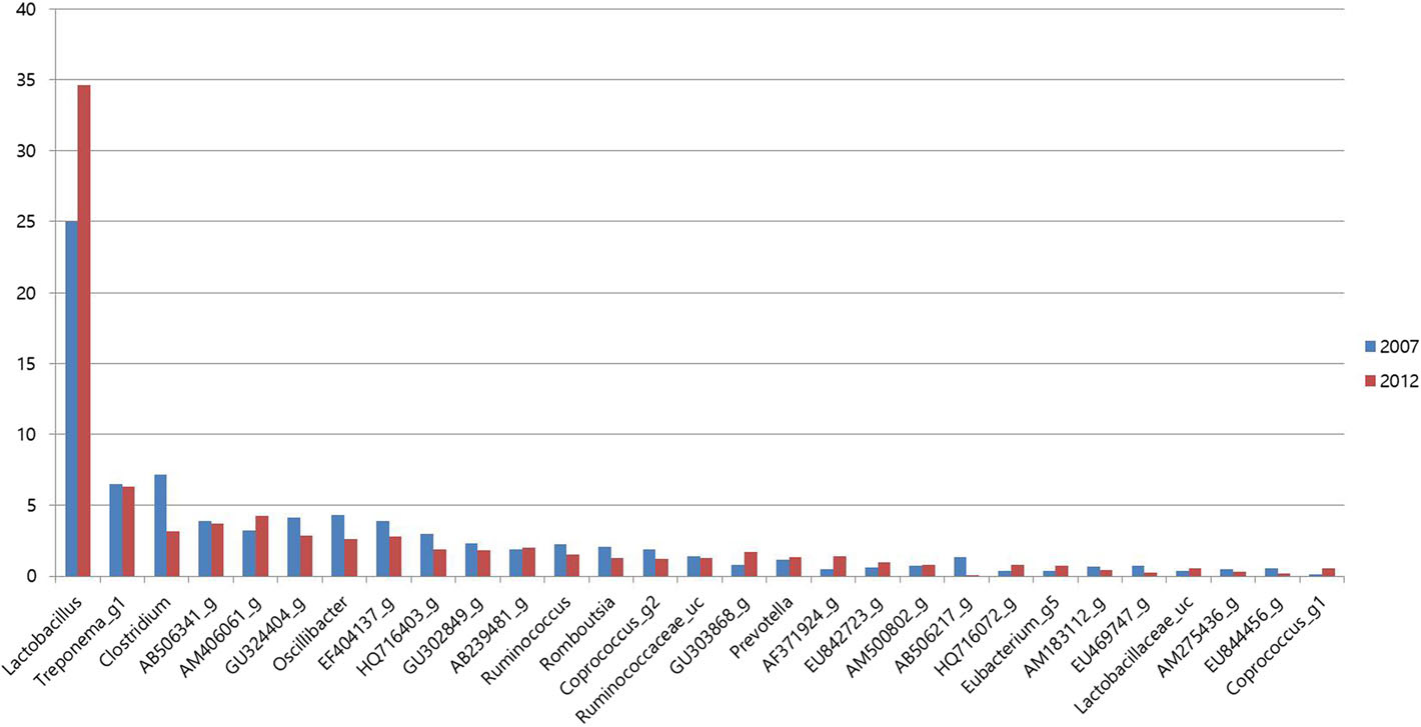
Bacterial taxonomic compositions of genus level^*^. ^*^Four samples from each treatment were analyzed for composition of genus level

Among the total genera detected, the bacterial genera including *Bacillus, Coprococcus, and Bacteriodes* were increased (*p* < 0.05), while *Oscillibacter, Clostridium, Ruminococcus, and Coprococcus* were less in 2012 group than in 2007 group Fig. 2. In species level, *Lactobacillus* species were increased (*P* < 0.05) in 2012 diet fed pigs than control. *Lactobacillus* species are well known to provide beneficial effects on gut function and health [49]. Moreover, the pathogenic bacteria *Clostridium* were reduced in 2012 group.

## Conclusion

These data well support our hypothesis that administration of pig diet formulated with ideal protein concept improves gut fermentation and healthiness, as well as helps minimize odorous compound generation from pig farm.

## Abbreviations

ADG: Average daily gain
ARC: Agriculture research council
BCFA: Branched chain fatty acids
BOD: Biochemical oxygen demand
CP: Crude protein
EDTA: Ethylenediaminetetraacetic acid
FAO: Food and Agriculture Organization
FCR: Feed conversion ratio
FID: Flame ionization detector
GC: Gas chromatography
GLM: Genernal linear model
INRA: Institute National de la recherche agronomique
NRC: National ResearchCouncil
OECD: Organisation for EconomicCo-operation and Development
OTUs: Operational taxonomic units
PCR: Polymerase chain reaction
SCFA: Short chain fatty acids
TKN: Total Kjeldahl nitrogen
VFA: Total volatile fatty acids
VOS: volatile odorous compounds
